# Case Report: Pregnant ROS1+ lung cancer patient treated with crizotinib - Impact on infancy

**DOI:** 10.3389/fonc.2026.1784859

**Published:** 2026-03-10

**Authors:** Theresa Weber, Jörg Hoffmann, Christian Michel, Andreas Burchert, Jelena Pesek, R. Verena Taudte, Katharina Schoner, Hilda Bartos, Simon Viniol, Paul Weiland, Andreas Neubauer, Sabine Flommersfeld, Siegmund Köhler, Mirjam Jung, Stefanie Weber, Svenja Hornig, Ines Wallot

**Affiliations:** 1 Marburg University, Department of Medicine, Marburg, Germany; 2 Department of Hematology, Oncology, and Immunology, University Hospital Giessen and Marburg, Marburg, Germany; 3 Core Facility for Metabolomics, Marburg, Germany; 4 Institute of Pathology, Fetal Pathology, University Hospital Giessen and Marburg, Marburg, Germany; 5 Clinic of Diagnostic and Interventional Radiology, University Hospital Giessen and Marburg, Marburg, Germany; 6 Center for Transfusion Medicine and Hemotherapy, University Hospital Giessen and Marburg, Marburg, Germany; 7 Department of Gynecology, Prenatal Medicine and Fetal Therapy, University Hospital Giessen and Marburg, Marburg, Germany; 8 Department of Pediatrics, University Hospital Giessen and Marburg, Marburg, Germany

**Keywords:** lung cancer, neonatology, pregnancy, ROS1, targeted therapy

## Abstract

Lung cancer remains the leading cause of cancer-related deaths worldwide. Managing cancer treatment during pregnancy is a rare yet challenging condition, with limited data available on maternal and neonatal outcomes. We present the case of a 37-year-old pregnant woman diagnosed with ROS-1-rearranged metastatic NSCLC, who was treated with crizotinib and subsequently delivered a preterm infant. Our report underscores the critical need for rigorous thromboembolic monitoring in pregnant patients undergoing cancer treatment. Furthermore, we provide evidence that placental tissue significantly reduces fetal crizotinib exposure, suggesting that crizotinib might be a viable therapeutic option for maintaining a pregnancy during lung cancer treatment.

## 1 Introduction

Lung cancer is the leading cause of cancer-related mortality worldwide (1). Proto-oncogene-tyrosine-protein-kinase-1 (ROS-1) rearrangements occur in approximately 2% of non-small-cell lung cancers (NSCLCs), with a notable prevalence in lung adenocarcinomas. These translocations are more frequently observed in women, non-smokers, and younger patients (1). Since 2016, crizotinib, an inhibitor of the c-Met/hepatocyte growth factor receptor tyrosine kinase that also blocks ROS-1 activity, has been established as the first-line therapy for ROS-1-translocated lung carcinomas, achieving response rates of approximately two-thirds and a median progression-free survival of 20 months before the emergence of drug resistance (1).

Here, we report a case of a 37-year-old woman diagnosed with ROS-1-translocated, metastatic NSCLC during pregnancy, who, despite her critical condition, opted to continue her pregnancy.

## 2 Case descriptions

### 2.1 The mother

A 37-year-old woman, never smoker, presented to her general practitioner (GP) with shortness of breath, dyspnea and cough. Her medical history included an emergency cesarian section due to premature placental abruption and polycystic ovarian syndrome, leading to an unfulfilled desire for a second child. Her very first pregnancy was without any complication. Other chronic illnesses were not reported, no alcohol or drugs were consumed.

Initially diagnosed with new-onset asthma, she was prescribed symptomatic treatment and bronchodilators. However, the symptoms persisted. On re-evaluation, she reported a weight loss of 20 kilograms over six months, prompting her referral to the hospital. A chest X-ray revealed a right hilar mass, raising suspicion of malignancy. Further imaging and laboratory tests confirmed liver metastases and a first trimester pregnancy. This was naturally conceived and the patient until then unaware of. There has never been a fertility treatment.

A left axillary lymph node biopsy confirmed metastatic, poorly differentiated, TTF-1 positive pulmonary adenocarcinoma (Ki-67 60%, TPS-Score 70%). Additionally, she developed unilateral right lower limb swelling. Ultrasound revealed a deep venous thrombosis extending from the calf muscle veins to the popliteal vein (>10 cm). Low molecular weight heparins (LMWH) anticoagulation monitored by anti-Xa assays was initiated, and a port catheter was implanted. There was no family history regarding cancer or thromboembolisms.

**Figure 1:**
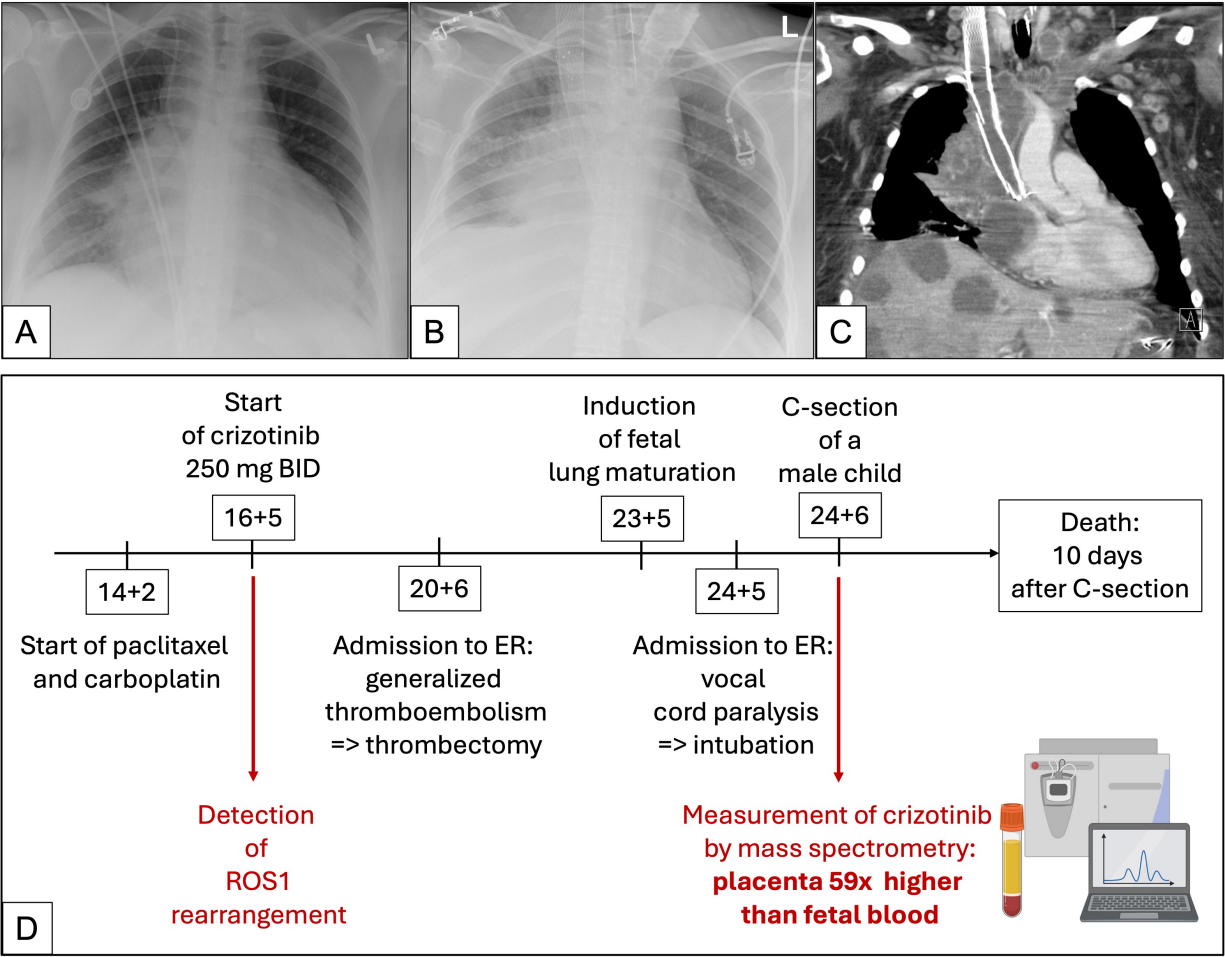
Timeline, X-rays and CT scans of the mother. **(A)** Chest-X-ray shows an enlarged cardiac silhouette conclusive with malignant pericardial effusion. Port catheter *in situ*. Right hilar mass. **(B, C)** X-ray and CT scan of the progressive lung disease after caesarian section and intubation. Exemplary depicted are new liver metastases, malignant lymphadenopathy, and stenting of the vena cava superior due to TE. **(D)** Timeline of disease progression. Partially created in BioRender.com [Publication License: Weiland, P. (2025) https://BioRender.com/k81h916. Arranged and finalized by Weber, T. (2025)]. ER, emergency room; C-section, caesarian section; BID, twice daily; x, times.

The patient was counseled about the pregnancy and possible conflict situations with the malignant disease and treatment options. The patient wished to continue the pregnancy. Before treatment could commence, the patient was urgently admitted with severe dyspnea, tachycardia (>135 bpm), diaphoresis, and respiratory distress requiring oxygen supply. Chest-X-ray and transthoracic echocardiogram revealed malignant pericardial effusion with increasing hemodynamic compromise (**Figure 1A**). Pericardiocentesis was performed, leading to immediate hemodynamic improvement. Given the high risk of recurrence, emergency chemotherapy with carboplatin and paclitaxel — considered tolerable during pregnancy (2) — was initiated at 14 + 2 weeks of gestation. Fetal viability was confirmed by prenatal ultrasound.

Shortly thereafter, molecular results identified a targetable ROS1 fusion. Given the limited data on ROS1-TKI use in pregnancy (3, 4), the patient and her family were counseled on off label treatment. She opted for crizotinib 250 mg BID, which was initiated at 16 + 5 weeks.

At 20 weeks, genetic thrombophilia screening (e.g. factor V Leiden mutation, prothrombin (G2021A) polymorphism) as well as antiphospholipid antibodies were negative, and the LMWH therapy was adjusted. D-dimer levels and factor VIII activity showed physiological ranges for a pregnancy.

However, shortly afterward, she developed superior vena cava syndrome with dyspnea, cyanosis, and right arm paresthesia. Imaging confirmed extensive thrombosis involving the right brachial, axillary, subclavian and internal jugular veins. The port catheter was removed, and anticoagulation was intensified. Due to persistent symptoms, an interventional thrombectomy with stent placement was performed. Fetal ultrasound revealed small-for-gestational-age (SGA) status (3rd percentile) with normal Doppler parameters. Given the maternal condition and uncertain prognosis, antenatal corticosteroids were administered at 23 + 5 weeks to accelerate fetal lung maturation.

At 24 + 2 weeks, the woman presented with severe dyspnea, inspiratory stridor, elevated respiratory rate, dyspnea, and a new-onset hoarseness. Laryngoscopy revealed vocal cord paralysis. Due to suspected progressive respiratory failure, she was intubated. Bronchoscopy confirmed tumor progression with trachea compression, necessitating stent placement. Given her critical condition, a multidisciplinary team recommended delivery on maternal indications. At 24 + 6 weeks, an extremely low birth weight male infant was delivered with APGAR scores of 3/2/2 and an umbilical artery pH of 7.22.

To quantify crizotinib exposure, we developed an in-house method based on liquid chromatography–mass spectrometry method. The fetal serum concentration was 11.2 nmol/L (= 10.9 fmol/mg assuming the density of serum by 1.03 g/ml (5)); while the placental tissue concentrations were markedly higher at 0.64 pmol/mg (= 640 fmol/mg), (see Appendix).

**Figure 2:**
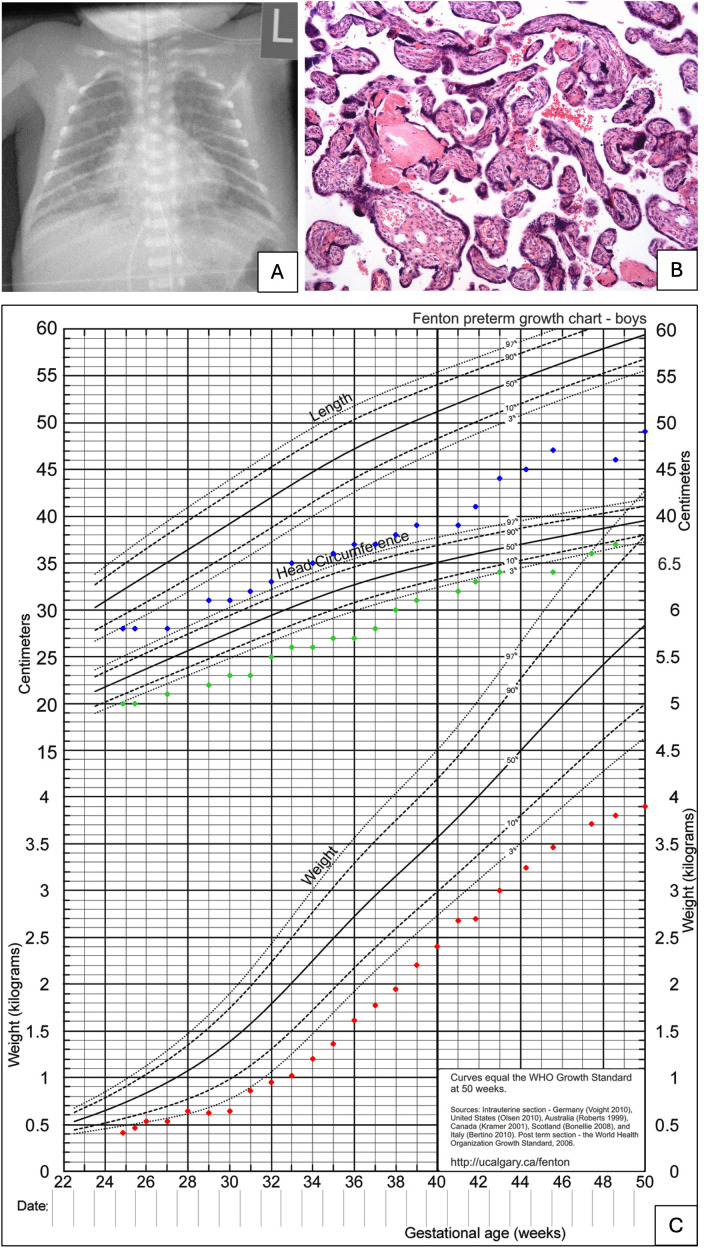
X-ray, percentiles of the child and placental histology. **(A)** X-ray of the infant at his first day of life. **(B)** Placental histology reveals accelerated maturation. Many terminal villi are extremely small, and trophoblastic nuclei of many villi are clustered, forming trophoblastic knots, so-called Tenney-Parker changes (Hematoxylin&Eosin x10). **(C)** Percentiles for weight (red dots), length (blue dots), and head circumference (green dots) from birth to 50 weeks. Discharge from hospital was at 53 weeks.

Histopathological examination of the placenta revealed severe growth restriction (<10th percentile for weight and basal area), villous maturation disorder and signs of long-lasting utero-placental hypoxia (**Figure 2B**). No maternal metastases and no signs of emboli or thrombi were detected.

Postpartum imaging in the mother showed progressive hepatic, splenic and pulmonary metastases alongside worsening thromboembolism, prompting another thrombectomy and further chemotherapy (**Figures 1B, C**).

Despite intensive care, the patient developed progressive metabolic acidosis and renal failure. requiring continuous veno-venous hemofiltration. Her condition deteriorated, with rising inflammatory markers indicating systemic infection. She passed away 10 days after cesarean section (**Figure 1D**).

### 2.2 The infant

The male preterm was delivered via caesarean section at 24 + 6 weeks of gestation, presenting as small for gestational age (SGA) with a birth weight of 410 g and a body length of 28 cm (1st/4th percentile) (**Figure 2C**).

Postnatal adaptation was complicated by bradycardia and severe respiratory insufficiency, requiring intubation within the first minutes of life. Respiratory stabilization was achieved only after intratracheal surfactant administration and high-frequency oscillatory ventilation. Chest-X-ray revealed stage III respiratory distress syndrome as well as signs of interstitial emphysema (**Figure 2A**).

Weaning from respirator was difficult and mechanical ventilation was challenging, so the infant required ventilatory support for the first five weeks of life.

However, following two courses of dexamethasone and additional diuretic therapy, extubation was successfully performed. After a prolonged period of non-invasive ventilation with NIPPV and CPAP, the infant was discharged at a corrected gestational age of 53 weeks on nasal high-flow therapy with a stable oxygen concentration of FiO2 0.23. The persistent respiratory impairment was accompanied by failure to thrive, necessitating nutritional supplementation with up to 150 kcal/kg/day. By discharge, the infant´s weight had reached the second percentile.

Abdominal ultrasonography detected a unilateral multicystic dysplastic kidney on the right side, while the left kidney appeared structurally normal with no clinical signs of renal dysfunction. Brain ultrasound findings were unremarkable. Retinopathy screening showed stage III retinopathy of prematurity (ROP), for which two intravitreal injections of the monoclonal antibody ranibizumab were administered.

In summary, preterm birth resulted in severe bronchopulmonary dysplasia (BPD) and possibly ROP-associated visual impairment, necessitating close follow up, including neurological development monitoring.

To further investigate the cause of small for gestational age (SGA) and the renal malformation, pathomorphological examination of the placenta as well as cytogenetic and molecular genetic analyses were conducted. Chromosome analysis revealed a normal male karyotype [46, XY]. A multigene panel analysis of 663 genes revealed no evidence of skeletal dysplasia or genetic variants associated with short stature or kidney malformations. Additional methylation studies were performed, which yielded unremarkable results.

A first follow-up at the age of seven months was conducted with neurodevelopmental assessment performed using the Bayley Scales of Infant and Toddler Development (BSID) III. At this point, the BSID results were below average results in all categories. Nevertheless, the boy showed promising progress in motor development. He was able to grasp a toy, transfer it between hands, and roll from his back to his stomach while supporting his upper body on his forearms. Additionally, he exhibited social and sensory responsiveness, smiling, vocalizing, and tracking stimuli with his eyes.

## 3 Perspectives of the family and clinical staff

The family had always expressed their deep wish to pursue the pregnancy. Even though, possible rapid worsening of the mother was discussed several times our patient and her family urged to proceed. Their own religious beliefs were strong, and the infant was long wished-for, so a termination of the pregnancy was out of question.

Thus, our interdisciplinary treatment team counseled the patient about the pregnancy, conflict situations with the malignant disease and treatment options for several times. Together, we searched a possible treatment path suitable for the mother and the child. All caring clinicians and nursing staff were notable emotionally involved in our patient’s story. Her death was mourned across the clinic. Hence, we decided together to give her and her little son a voice by writing this report.

Clinicians treating patients in similar situations will be confronted with lots of emotionally disturbing and clinically challenging situations. Team members might be of different opinions also regarding the patient’s decision. Though, we as interdisciplinary treatment team had the obligation to respect the patient’s wishes and to chaperone her as good as possible and as needed.

The patient’s family and herself never showed any doubts to proceed the pregnancy. The boy was born as a high risk extremely low birth weight preterm with several associated morbidities after birth which must be closely monitored in the future. But there is a strong network of family and community members which support the father, the older daughter and the beloved small boy until today. This strong coherence was impressive at any time.

## 4 Discussion

With increasing maternal age in society, physicians will more often encounter with cancer during pregnancy. Both pregnancy and malignancies are associated with a heightened risk of thromboembolic events (TE).

Similarly, ROS1-translocations are known to increase TE risk, negatively impacting survival rates (1, 6, 7). The median progression-free survival (PFS) after first-line ROS1-TKIs is significantly longer in patients without TE compared to those with TE (26 months vs. 12 months) (8).

Our patient experienced multiple TE, suggesting a multiplied risk of TE in ROS-1 translocated NSCLC accompanied by pregnancy. Therefore, close monitoring is essential, and additional risk factors (e.g. catheters, cortisone, changes in antithrombotic medication) should be avoided if possible.

For the first time, we measured crizotinib levels in placenta tissue following maternal crizotinib therapy during pregnancy, as well as in neonatal blood samples. The concentration in the placenta was 59 times higher than in the fetal serum, consistent with ex vivo dual-side perfusion studies of human cotyledons of a healthy, full-term placenta (9). These findings indicate that placental tissue accumulates crizotinib *in vivo* thereby limiting fetal exposure to high drug levels.

To our knowledge, only two cases of pregnant women receiving crizotinib have been reported in the literature (4, 10). In both cases, infants were delivered preterm (at 30 weeks and 26 + 2 weeks of gestation, respectively) but exhibited normal development. Based on these case reports, we decided together with the patient to start crizotinib.

In our case, the pregnancy and the malignancy were diagnosed almost simultaneously in the first trimester. Despite the mother’s poor prognosis, crizotinib treatment enabled continuation until fetal viability, in accordance with the parents’ wishes.

The dosing regimen of crizotinib remains a challenging question as it is mainly metabolized in the liver. Hence, drug metabolism might be affected due to pregnancy related changes (11). Additionally, immunological alterations during pregnancy might affect cancer initiation itself and supports its growth during pregnancy by shared mechanisms of e.g. immune tolerance (12). Potential cancer promoting factors of pregnancies and their impacts on anti-cancer drug regimens remain largely unknown showing utmost importance of patient-centered monitoring and further research.

Although the neonate was born as an extremely-low-birth-weight preterm infant, he did not develop severe complications apart from BPD, which required prolonged oxygen therapy, and stage III ROP. His neurological prognosis appears positive. With physiotherapeutic support during the hospitalization and subsequent outpatient care, his developmental trajectory has remained favorable.

Genetic testing including whole-exome sequencing, revealed no abnormalities. Given that DNA methylation is an epigenetic mechanism influenced by chemotherapy (13) and cancer itself (14), we additionally analyzed the methylation status of 13 gene loci associated with growth retardation (ANKRD11, CREBBP, EP300, FAM50A, FANCA, KDM3B, KDM5C, KDM6A, KMT2A, KMT2D, PQBP1, SIN3A, and SRCAP) but no abnormalities were detected. Therefore, fetal growth restriction is most likely attributable to histopathologically confirmed placental dysfunction.

Whether the placental pathology resulted from maternal thromboembolic complications and/or crizotinib therapy (e.g. hypoxic and/or toxic chorionic tissue damage) remains unclear.

However, our findings suggest that the placenta provides a protective barrier, preventing excessive fetal crizotinib exposure. To date, no severe complications or teratogenic effects have been reported following *in utero* crizotinib exposure. However, given the limited number of cases, definitive conclusions cannot yet be drawn. We encourage further case reports, particularly to assess teratogenicity and malformation risk, as congenital anomalies have been observed with other tyrosine kinase inhibitors (15).

## 5 Conclusions

Pregnant women receiving crizotinib should undergo close monitoring for thromboembolic events, as well as for fetal development and placental function. Despite these considerations, crizotinib may be a viable option for maintaining a pregnancy during lung cancer treatment. To validate our findings on crizotinib concentrations in placental tissue and neonatal serum, further analyzes should be conducted in future cases.

## Data Availability

The original contributions presented in the study are included in the article/supplementary material. Further inquiries can be directed to the corresponding authors.

## Appendix

### 1.1 Method of crizotinib quantification by LC-MS

Crizotinib was measured by LC-MS, using an Agilent 1290 HPLC coupled to a QTrap 5500 mass spectrometer (AB Sciex).

100 µl of serum was mixed with 10 µl internal standard (Crizotinib-d5; 0.1 µM) and 400 µl methanol. After centrifugation the supernatant was dried at 4 °C over night (Labconco Centrivap Concentrator) and the residue subsequently dissolved in 100 µl 10% methanol, containing 0.1% formic acid.

For placenta tissue 50–100 mg was mixed with 10 µl internal standard (10 µM) and 900 µl methanol. Samples were homogenized by using two stainless steel beads (2.8 mm) and a homogenizer (10 cycles of 30 s at 4 °C with 15 s pause in between; Bead Blaster 24R, Benchmark). The residue of the dried supernatant was dissolved in 1 ml methanol.

The LC-MS injection volumes were 10 µl for serum and 1 µl for tissue samples. Separation took place on a Kinetex reversed-phase C18 column (2.1 × 50 mm; Phenomenex) using 5% and 100% methanol as solvents A, and B respectively. To each solvent was added 0.1% formic acid.

The gradient was as follows: 0 min (10% B), 7 min (100% B), 11 min (100% B), 11.1 min (10% B) and 15 min (10% B). The flow rate was 0.3 ml min-1.

Compounds were detected in multiple reaction monitoring mode (positive ionization) by tracing the following transitions: Crizotinib 450 → 260, 177 and Crizotinib-d5 455 → 265, 178.

For quantification a respective 4-point calibration curve was used for each sample type (serum: 2–20 nM and tissue: 20–200 nM). Calibration samples were prepared by spiking the appropriate amount of crizotinib in drug free serum and tissue, respectively.

Data analysis was performed using Analyst 1.7.2 and MultiQuant 2.1.1 (AB Sciex). Crizotinib and Crizotinib-d5 were purchased from Biozol Diagnostica (Eching, Germany) and Biomol GmbH (Hamburg, Germany), respectively.
